# Spontaneous left main coronary artery dissection occurred in a young male: a case report and review of literature

**DOI:** 10.1186/s12872-022-02642-5

**Published:** 2022-06-07

**Authors:** Yongjun Li, Mingming Yang, Xi Chen, Xiaoguo Zhang, Rui Zhang, Pengfei Zuo, Lei Jiang, Genshan Ma

**Affiliations:** 1 Department of Cardiology, Zhongda Hospital, School of Medicine, Southeast University, 87 Dingjiaqiao, Nanjing, 210009 People’s Republic of China; 2 Wandong People Hospital, Chuzhou, 239000 Anhui People’s Republic of China

**Keywords:** Spontaneous coronary artery dissection, Left main coronary artery, Acute myocardial infraction, Case report

## Abstract

**Background:**

Spontaneous coronary artery dissection (SCAD) is now recognized as an important cause of acute coronary syndrome (ACS), which is thought to be more prevalent in women. However, the male patients, on the other hand, cannot be easily ignored.

**Case presentation:**

A 26-year-old male suffered from SCAD that occurred in the left main coronary artery (LMCA) and a secondary acute myocardial infraction (AMI). Coronary CT angiography and coronary angiography (CAG) revealed aneurysms in the LMCA and right coronary artery (RCA), as well as a total occlusion in the proximal branch of the left anterior descending artery (LAD). Along with drug therapy, coronary artery bypass graft (CABG) surgery was recommended, and the patient has been symptom-free for one year.

**Conclusion:**

We report a case of spontaneous left main coronary artery dissection that occurred in a young male. The necessity of identifying typical imaging features and following up patients with SCAD for life to reduce the risk of fatal cardiac complications cannot be overstated.

## Background

Spontaneous coronary artery dissection (SCAD), originally considered to be a rare cause of acute coronary syndrome (ACS), is now recognized as an important cause of myocardial infarction (MI) as this condition is increasingly and accurately diagnosed [1]. SCAD can occur in any coronary artery, with the left anterior descending artery (LAD) being the most frequently involved, followed by the right coronary artery (RCA) and its branches, and rarely occurring in the left main coronary artery (LMCA). We successfully cured a young man with SCAD in LMCA, resulting in a large area of MI, which was relatively rare in clinical diagnosis and treatment. This paper aims to sort out and present the clinical imaging and pathological data of this patient in order to provide ideas for the diagnosis and treatment of patients with MI caused by SCAD.

## Case presentation

**Fig. 1:**
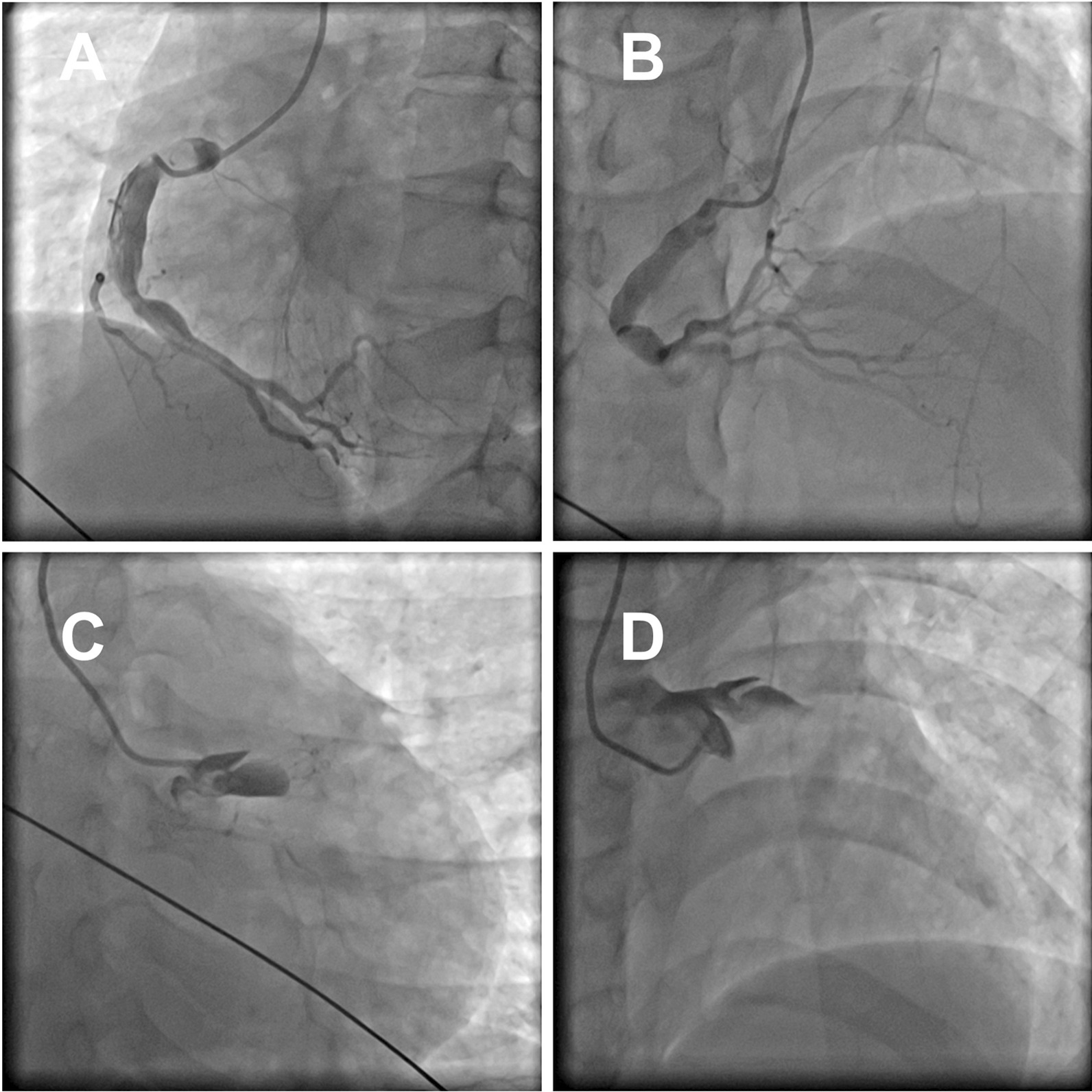
Emergency coronary CT angiography showing right coronary dilatation, spontaneous left main dissection

A 26-year-old male patient presented with sudden-onset chest pains, each lasting for several hours, that remained untreated for a half month. Nine hours after the pain intensified, he was referred to the local hospital for treatment. The electrocardiogram (ECG) showed ST-segment elevation in the AVR lead and moderate ST-segment depression in other leads. Emergency coronary angiography (CAG) showed coronary heart disease (CHD), acute myocardial infarction (AMI), and spontaneous left main dissection (Fig. 1). Based on the existing inspection results, the patient was diagnosed with AMI and spontaneous dissection of the LMCA. Given the critical condition, the patient was transferred to the department of cardiology at our institution for further diagnosis and treatment.

**Fig. 2:**
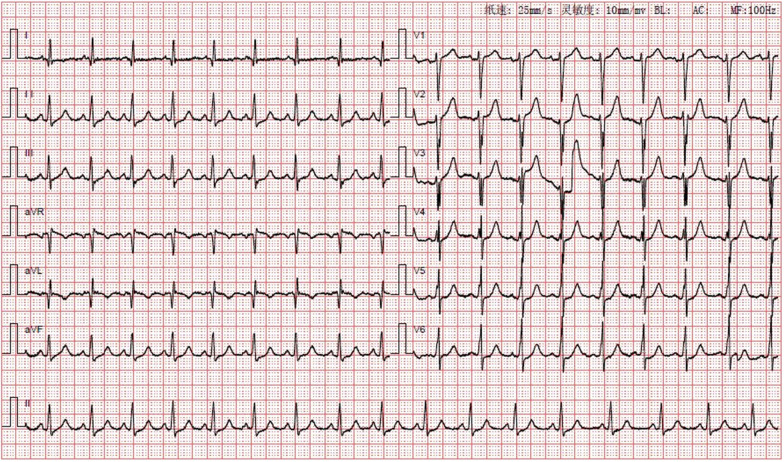
Electrocardiogram (ECG) showing sinus tachycardia with abnormal Q waves

**Fig. 3:**
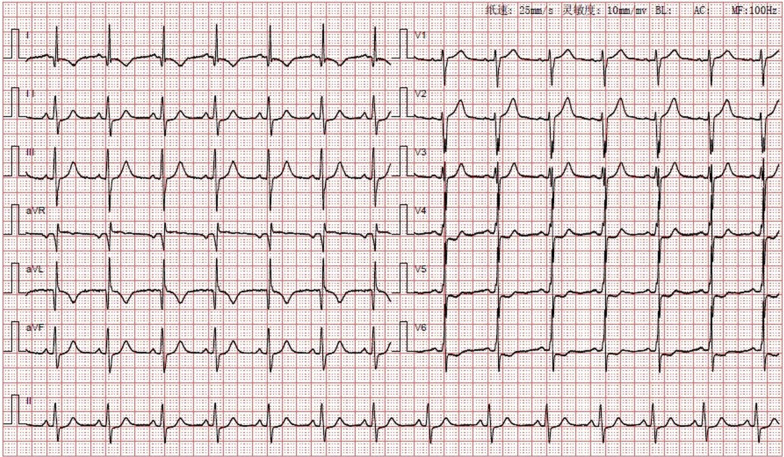
Re-examination of the ECG showing an elevated right ventricular voltage and an abnormal ST-T segment

**Fig. 4:**
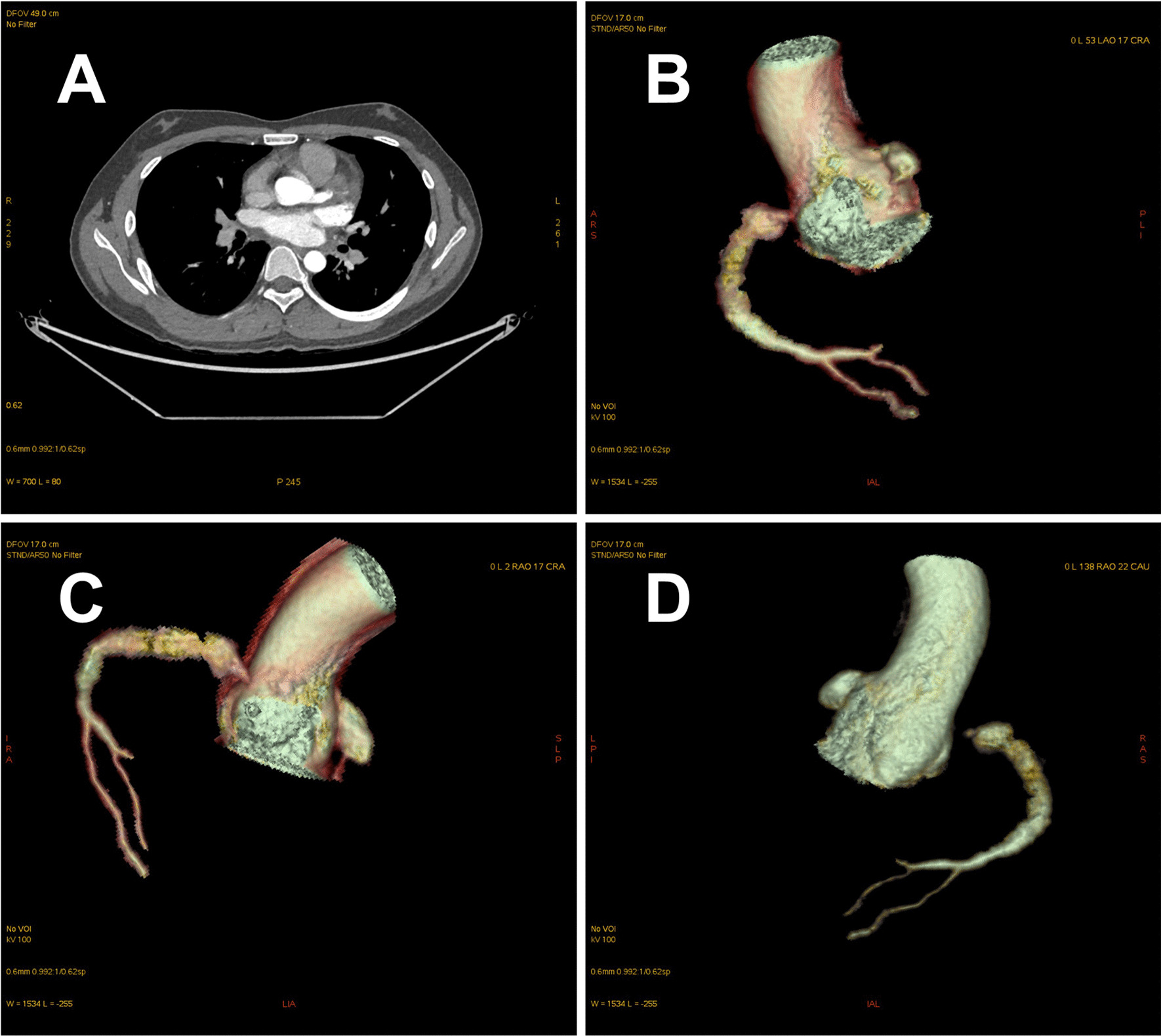
Coronary CT angiography showing the presence of aneurysms in left main coronary artery (LMCA) and right coronary artery (RCA)

**Fig. 5:**
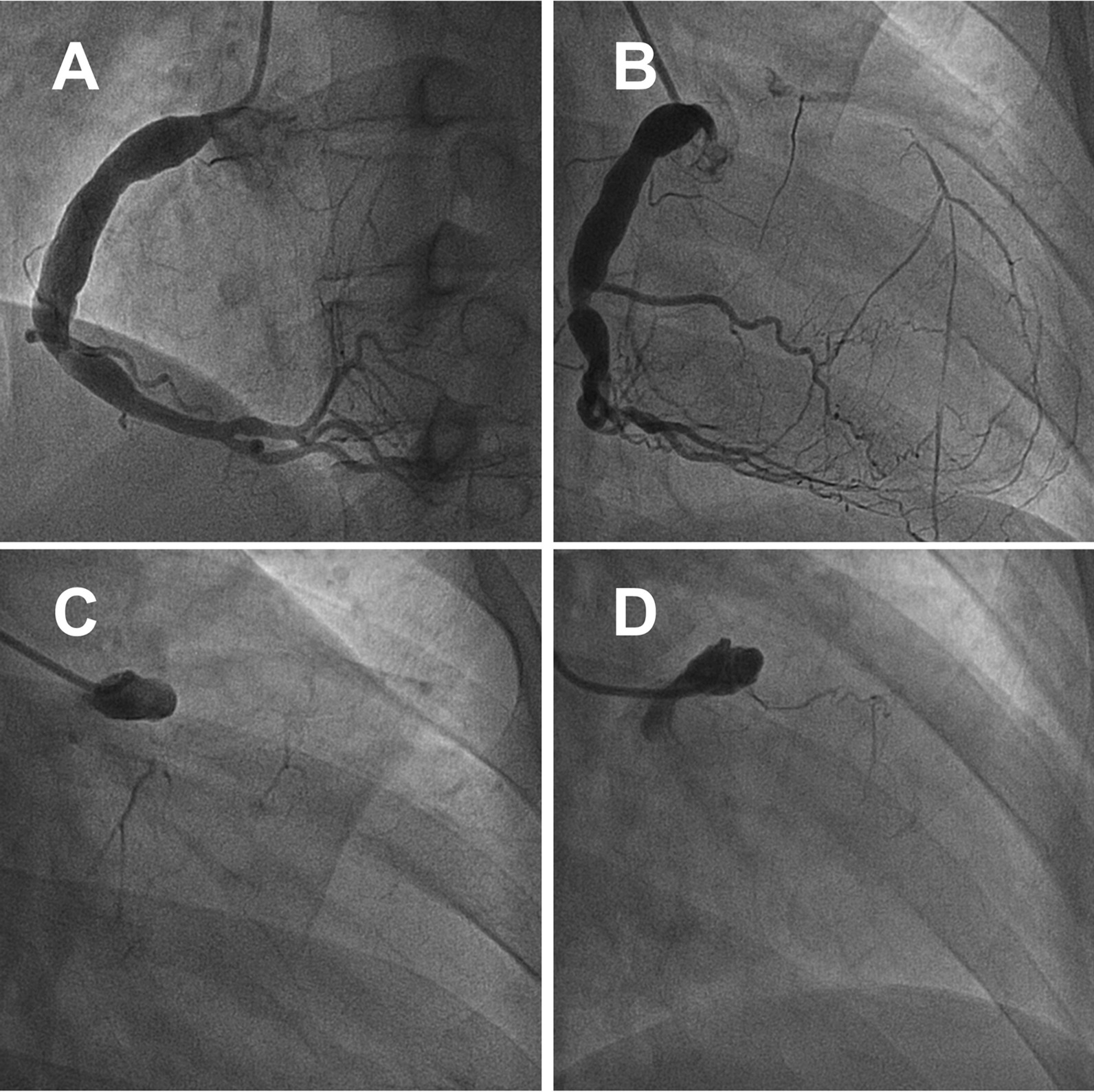
Coronary angiography (CAG) showing total occlusion in the proximal branch of the left anterior descending artery (LAD)

On admission, the blood pressure of the patient was 83/49 mmHg, with no significant difference between the arms, and heart rate was 65 beats per minute. Peripheral arteries were equally palpable, and there were no vascular bruits or heart murmurs. The patient was healthy and had no history of smoking. The ECG showed sinus tachycardia with abnormal Q waves (Fig. 2). The laboratory examination showed that the white blood cell count was 20.49*10^9/L, the neutrophil ratio was 94.5%, the creatinine kinase isoenzyme and cardiac troponin I were 189 ng/ml and 1.7 ng/ml, respectively. There was no abnormality in the blood lipid profile. The echocardiography revealed segmental left ventricular wall motion abnormalities, a low ejection fraction of 39%, and a modest amount of pericardial effusion. During hospitalization, myocardial necrosis biomarkers elevated to 28.56 ng/ml for cardiac troponin I and 2010 pg/ml for NT-proBNP. Re-examination of the ECG after two days of admission showed an elevated right ventricular voltage and an abnormal ST-T segment (Fig. 3). Coronary CT angiography showed aneurysms in LMCA (diameter, 8 mm) and RCA (diameter, 9 mm) (Fig. 4). CAG indicated complete occlusion in the proximal branch of the LAD (Fig. 5). Based on the features of the symptoms, the patient was diagnosed CHD, AMI, spontaneous left main dissection, and right coronary dilatation. The involved location was LMCA, which led to AMI. Since being admitted to our hospital, the patient had had repeated chest tightness and pain, and the situation was very serious. Upon a week of symptomatic treatment, such as aspirin anti-aggregation, heparin sodium anticoagulation, coronary vasodilation, lipid management, and diuresis, the patient’s condition improved. After that, the patient underwent off-pump coronary artery bypass graft surgery (OPCABG). The necessity of following up the patients with SCAD for life was fully informed to patient before discharge. The patient has been symptom-free for one year.

## Discussion and conclusions

Although SCAD has been recognized as one of the significant causes of AMI, the real prevalence and etiology remain unknown [2]. At present, it is believed that SCAD occurs primarily in young to middle-aged women, particularly those who are pregnant or breastfeeding with low cardiovascular risk factors, possibly due to female sex hormones [3, 4]. Furthermore, connective tissue diseases (Marfone syndrome, Ehlers-Danlos syndrome), vasculitis (polyarteritis nodosa, systemic lupus erythematosus, and Kawasaki disease), atherosclerosis, and fibromuscular dysplasia are considered risk factors for SCAD. Strenuous activities, acute hypertension, and intense mental stress may also contribute to SCAD [5, 6].

In this case, the patient is a young man who is usually in good physical condition, has no bad habits, no classic risk factors, and no family history of coronary heart disease (CHD). Autoimmune diseases were excluded during the hospitalization. Combining with the characteristics of coronary artery diseases, it was found to be highly consistent with the coronary artery diseases caused by Kawasaki disease (KD). We postulate that his SCAD was induced by KD, although no KD-related clinical data or history was provided.

KD is an inflammatory disease that attacks systemic small and medium blood vessels and usually occurs in children between 6 months and 5 years old. The diagnosis of KD lacks specific laboratory tests and gold standards, and mainly depends on the clinical symptoms of the child [7, 8]. Children with typical KD who are diagnosed and treated in a timely and effective manner possess a good prognosis [9]. However, children with atypical KD may only have atypical symptoms like fever or rash, and always fail to receive timely and effective treatment due to missed diagnosis, misdiagnosis, or delayed diagnosis, which can lead to the involvement of multiple organs, especially cardiovascular complications [8, 10].

Coronary artery lesions (CALs) are considered the most serious complications of KD [11]. The long-term outcome of vasculopathy in KD has not been well clarified. More and more clinical evidence indicates that the sequelae of CALs will persist into adulthood [12]. A retrospective study in Japan found that compared with ACS patients, patients with a history of KD will develop AMI when the coronary risk score is lower and the age of onset is younger [13]. Coronary arteritis caused by KD is one of the causes of atherosclerosis in young adults and one of the risk factors for acute coronary events [14, 15].

In the present case, the obvious involvement of the coronary artery includes not only coronary artery aneurysm and right coronary artery dilatation, but also spontaneous dissection of the LMCA and even the occurrence of AMI. The condition is critical and rare, and was considered to be related to coronary vasculitis caused by KD. We speculate that the patient may have suffered from atypical KD as a child but did not receive timely and effective treatment, resulting in the damage caused by the KD to the coronary artery being ignored. We assume that the AMI and SCAD of the LMCA and other adverse cardiac events in the patient can be ascribed to the cardiac sequelae of KD.

Regardless of the cause of the SCAD in LMCA and the occurrence of AMI, it is currently considered that conservative therapy and drug therapy (antiplatelet therapy and anticoagulant drugs) are preferred strategies. Meanwhile, intravenous fibrinolysis should be vigilant as it has the potential to exacerbate the dissection or hematoma, worsen the condition, and even result in mortality [16]. However, percutaneous coronary intervention (PCI) or coronary artery bypass grafting (CABG) may be preferred if the patient is hemodynamically unstable or has persistent ischemia or recurrent dissection [17, 18]. In short, personalized treatment is required for each patient’s specific conditions, as is long-term follow-up to monitor the prognosis. Following admission, this patient suffered repeated chest tightness and pain, and the involved location was LMCA, which led to the patient's serious condition. Finally, conservative treatment (aspirin anti-aggregation, heparin sodium anticoagulation, coronary dilatation, ventricular rate, and blood pressure control) was chosen till his condition was stable and CABG was performed. He recovered and has been followed up since discharge, and no discomfort, such as chest tightness or pain, has occurred again. The follow-up will be continued in the future. Although recent evidence has shed light on the natural history and management of SCAD, the real causes of SCAD remain unclear and there is no consensus on clinical treatment. Therefore, more attention should be drawn to SCAD and further research needed to be conducted in order to better understand and treat patients with SCAD.

In conclusion, we reported a case of a 26-year-old male patient with SCAD of LMCA and secondary AMI. Although SCAD is an extremely rare disease and challenging, it is becoming increasingly recognized and diagnosed. Physicians and adult cardiologists should strengthen their understanding of SCAD, which may help to lower the incidence of severe complications such as ACS and sudden death.

## Data Availability

Data are available from Yongjun Li (email: liyongjunnj@hotmail.com) upon reasonable request and with permission by Zhongda Hospital and Wandong People Hospital.

## Abbreviations

SCAD: Spontaneous coronary artery dissection
ACS: Acute coronary syndrome
LMCA: Left main coronary artery
AMI: Acute myocardial infraction
CAG: Coronary angiography
RCA: Right coronary artery
LAD: Left anterior descending artery
CABG: Coronary artery bypass graft
ECG: Electrocardiogram
KD: Kawasaki disease
OPCABG: Off-pump coronary artery bypass graft surgery
CHD: Coronary heart disease
CAL: Coronary artery lesion
PCI: Percutaneous coronary intervention (PCI)
CABG: Coronary artery bypass grafting
MI: Myocardial infarction

## References

[1] Adlam D, García-Guimaraes M, Maas A (2019). Spontaneous coronary artery dissection: no longer a rare disease. Eur Heart J.

[2] Hayes SN, Tweet MS, Adlam D, Kim ESH, Gulati R, Price JE, Rose CH (2020). Spontaneous coronary artery dissection: JACC state-of-the-art review. J Am Coll Cardiol.

[3] Tweet MS, Gulati R, Williamson EE, Vrtiska TJ, Hayes SN (2016). Multimodality imaging for spontaneous coronary artery dissection in women. JACC Cardiovasc Imaging.

[4] Díez-Villanueva P, García-Guimaraes MM, Macaya F, Masotti M, Nogales JM, Jimenez-Kockar M, Velázquez M, Lozano Í, Moreu J, Avanzas P (2021). Spontaneous coronary artery dissection and menopause. Am J Cardiol.

[5] Saw J, Humphries K, Aymong E, Sedlak T, Prakash R, Starovoytov A, Mancini GBJ (2017). Spontaneous coronary artery dissection: clinical outcomes and risk of recurrence. J Am Coll Cardiol.

[6] Saw J, Aymong E, Sedlak T, Buller CE, Starovoytov A, Ricci D, Robinson S, Vuurmans T, Gao M, Humphries K (2014). Spontaneous coronary artery dissection: association with predisposing arteriopathies and precipitating stressors and cardiovascular outcomes. Circ Cardiovasc Interv.

[7] Dietz SM, van Stijn D, Burgner D, Levin M, Kuipers IM, Hutten BA, Kuijpers TW (2017). Dissecting Kawasaki disease: a state-of-the-art review. Eur J Pediatr.

[8] McCrindle BW, Rowley AH, Newburger JW, Burns JC, Bolger AF, Gewitz M, Baker AL, Jackson MA, Takahashi M, Shah PB (2017). Diagnosis, treatment, and long-term management of Kawasaki disease: a scientific statement for health professionals from the American heart association. Circulation.

[9] Friedman KG, Gauvreau K, Hamaoka-Okamoto A, Tang A, Berry E, Tremoulet AH, Mahavadi VS, Baker A, deFerranti SD, Fulton DR et al. Coronary artery aneurysms in Kawasaki disease: risk factors for progressive disease and adverse cardiac events in the US population. J Am Heart Assoc. 2016;5(9). 10.1161/JAHA.116.003289 PMC5079009 27633390

[10] Newburger JW, Takahashi M, Burns JC (2016). Kawasaki disease. J Am Coll Cardiol.

[11] Kuo HC (2017). Preventing coronary artery lesions in Kawasaki disease. Biomed J.

[12] Brogan P, Burns JC, Cornish J, Diwakar V, Eleftheriou D, Gordon JB, Gray HH, Johnson TW, Levin M, Malik I (2020). Lifetime cardiovascular management of patients with previous Kawasaki disease. Heart.

[13] Mitani Y, Tsuda E, Kato H, Higaki T, Fujiwara M, Ogawa S, Satoh F, Nakamura Y, Takahashi K, Ayusawa M (2019). Emergence and characterization of acute coronary syndrome in adults after confirmed or missed history of Kawasaki disease in Japan: a Japanese nationwide survey. Front Pediatr.

[14] Hartopo AB, Setianto BY (2013). Coronary artery sequel of Kawasaki disease in adulthood, a concern for internists and cardiologists. Acta Med Indones.

[15] Rizk SR, El Said G, Daniels LB, Burns JC, El Said H, Sorour KA, Gharib S, Gordon JB (2015). Acute myocardial ischemia in adults secondary to missed Kawasaki disease in childhood. Am J Cardiol.

[16] Saw J, Starovoytov A, Humphries K, Sheth T, So D, Minhas K, Brass N, Lavoie A, Bishop H, Lavi S (2019). Canadian spontaneous coronary artery dissection cohort study: in-hospital and 30-day outcomes. Eur Heart J.

[17] Al Mahruqi G, Alsabti H, Mukaddirov M (2021). Surgery is an option in evolving myocardial infarction induced by spontaneous coronary artery dissection: a case report. Eur Heart J Case Rep.

[18] Inohara T, Saw J, Kohsaka S, Fukuda K, Fushimi K (2020). Treatment pattern and outcome of spontaneous coronary artery dissection in Japan. Int J Cardiol.

